# Clinical Pathology of the Blood

**Published:** 1901-11

**Authors:** 


CLINICAL PATHOLOGY OF THE BLOOD. A treatise on the general principles and special applications of Hematology. By JAMES EWING, M. D., Professor of Pathology in Cornell University Medical College, New York City. In one handsome octavo volume of 432 pages, with 28 engravings and 14 full-page plates in colors. Just ready. Philadelphia and New York: Lea Brothers & Co. 1901. [Price, cloth, $3.50 net.]

The rapid advances in the knowledge of the pathology of the blood and the application of this knowledge in clinical diagnosis is the reason the author gives for the preparation of the present volume. He has striven and succeeded in giving a practical account of the changes in the blood and the relation between these changes and morbid states of the system, and the bearings on diagnosis and treatment are obvious and direct. Much of the information contained in this book is inaccessible to the general reader, being found only in monographs, or in hospital reports; these the author has sifted and the information thus secured has been incorporated in the respective chapters.

Ewing divides his work into VI. parts with an introduction on the interpretation of analysis of the blood, and advises that "the results of examination are to be interpreted only in the light of the fullest possible clinical information." Chapter I. deals with the general physiology and pathology of the blood, describing technics, chemistry, morphology and physiology of the red cells, the leukocytes, and the development of the blood cells. Part II. deals with the blood diseases, such as chlorosis, leukemia, progressive pernicious anemia, and pseudo-leukemia. Part III. takes up the acute infectious diseases; Part IV. the constitutional diseases; Part V. the general visceral diseases, and Part VI. the animal parasites. The features of the book are the beautiful full-page colored plates, 14 in number and the surprisingly complete list of references to the bibliography at the end of each chapter,—features well worth the price of the book alone. The book is well bound and printed. W. C. K.

